# Retrospective Study of Severe Atopic Disease in Young Children (0–5 Years) Managed with Dupilumab Highlights Significant Comorbidities

**DOI:** 10.3390/children12121639

**Published:** 2025-12-02

**Authors:** Evelyn F. Fagan, Elena B. Hawryluk, LaDonya Jackson-Cowan

**Affiliations:** 1 Department of Medical Education, Mercer University School of Medicine, Savannah, GA 31404, USA; 10986528@live.mercer.edu; 2 Department of Dermatology, Massachusetts General Hospital, Boston, MA 02114, USA; ehawryluk@mgh.harvard.edu; 3 Department of Dermatology, University of Miami, Miami, FL 33136, USA

**Keywords:** pediatric dermatology, atopic dermatitis, dupilumab, comorbidities, inter-specialty collaboration

## Abstract

**Highlights:**

**What are the main findings?**

- A substantial proportion of atopic dermatitis patients eligible for dupilumab have coexisting neurodevelopmental disorders.
- Coordinated care and inter-specialty collaboration are important for ensuring timely access to dupilumab, particularly for patients with multiple atopic conditions.

**What are the implications of the main findings?**

- Dermatologists should consider coexisting comorbidities when treating atopic dermatitis and expand the evaluation of dupilumab’s benefits beyond skin improvement.
- Inter-specialty collaboration should be encouraged to optimize access to dupilumab for young children.

**Abstract:**

Background: Atopic dermatitis (AD) is a common pediatric skin disease that is associated with other atopic comorbidities, all of which correlate with higher rates of neurocognitive alterations such as developmental delays and ADHD. Methods: We conducted a retrospective chart review from January 2022 through January 2024 and identified 79 children aged 0–5 years who were prescribed dupilumab in the Massachusetts General Brigham healthcare system. We defined the patient population (including demographics, atopic comorbidities, and neurocognitive burden), and assessed whether time to treatment access varied by patient or prescriber characteristics. Results: The mean age at dupilumab initiation was 3.4 years, and 62.0% of patients were male. The cohort was diverse (48.1% White, 25.3% Black, 16.5% Hispanic/Latino, 10.1% Asian/other), with 48.1% publicly insured. Atopic comorbidities were common: 64.6% had food allergies, 34.2% had asthma, and 10.1% had eosinophilic esophagitis (EOE); 73.4% had two or more atopic diagnoses. Neurodevelopmental disorders affected 43.0% of patients, with speech and language delay most frequent (25.3%) and higher rates among those with EOE (87.5% vs. 38.0%, *p* < 0.01). The mean time to dupilumab approval was 20.9 days, with dermatologists achieving faster approvals than other specialists (10.6 vs. 51.9 days, *p* < 0.001). Conclusions: Our findings reveal infrequently reported high rates of atopic and neurologic comorbidities in young children with AD and underscore the importance of coordinated inter-specialty collaboration to ensure timely access to dupilumab for these patients.

## 1. Introduction

Atopic dermatitis (AD) is a chronic, pruritic inflammatory skin disease that typically manifests during early childhood, impacting approximately 15% of children in the United States, including at least 2% with moderate-to-severe disease [1]. Among affected children, 45% develop AD within the first 6 months of life, 60% within the first year of life, and 85% by the age of 5 years [2]. Beyond its physical symptoms, AD significantly affects quality of life for both patients and their families. Children often experience disrupted sleep, chronic itching, and discomfort, leading to behavioral issues, concentration difficulties, and emotional dysregulation [3,4]. Parents report heightened stress, emotional exhaustion, and even suicidal ideation when managing severe pediatric AD [5]. The social and emotional toll of the disease can isolate families, interfere with routines, and damage caregiver–child bonding [6].

AD is also associated with numerous comorbidities, particularly other atopic conditions. It is typically the first manifestation in what is known as the “atopic march,” a well-established progression of allergic diseases that often begins with AD in infancy and is followed by the development of food allergies, asthma, allergic rhinitis, and eosinophilic esophagitis (EOE) [7]. These conditions frequently co-occur, and their overlapping pathophysiology pertaining to Th2 overexpression suggests a shared underlying immune dysregulation that begins early in life.

Importantly, recent studies have shown that children with AD, particularly those with early-onset or more severe disease, are at increased risk for neurodevelopmental disorders. These include autism spectrum disorder, developmental delays, memory impairment, speech and language impairments, seizures/epilepsy, and attention-deficit/hyperactivity disorder (ADHD) [8,9]. While the precise mechanisms remain under investigation, chronic systemic inflammation, sleep disruption, and stress responses likely contribute to altered brain development and cognitive functioning in this population [10]. Despite the recognized association between AD and a range of neurocognitive conditions, few studies have specifically characterized the epidemiology of these comorbidities in young children, particularly those with moderate-to-severe AD requiring biologic therapy. Existing research has primarily focused on ADHD, with studies demonstrating that children with atopic conditions are at increased risk and, in some cases, that AD may precede ADHD onset by several years [11,12]. While this emphasis on ADHD has advanced understanding of one important comorbidity, other neurodevelopmental outcomes, such as speech and language impairments, motor delays, and learning disabilities, remain less well characterized in the context of pediatric AD.

The pathogenesis of AD is driven largely by Th2 pathway activation, with interleukin (IL)-4 and IL-13 as central cytokines. While these molecules contribute to immune dysregulation and skin barrier impairment, they are also involved in normal neurodevelopment, where balanced signaling supports healthy brain maturation. Disruption of this balance in early life has been proposed as one possible link between AD and increased neurocognitive comorbidities [9].

Dupilumab, a targeted biologic treatment for AD, is indicated for patients with refractory moderate-to-severe disease and was approved for children over 6 months of age in 2022. It works by inhibiting interleukin-4 (IL-4) and interleukin-13 (IL-13), two cytokines central to the Th2-driven inflammation that underlies not only AD, but also many other allergic conditions.

By targeting this shared immunologic pathway, dupilumab has demonstrated therapeutic effects across multiple atopic diseases. In addition to reducing AD severity, clinical trials and real-world studies have shown that dupilumab can improve control of asthma symptoms, decrease exacerbation rates, and reduce corticosteroid dependence in patients with comorbid asthma [13]. It has also shown promise in improving eosinophilic esophagitis (EOE), leading to symptom reduction and histologic improvement [14]. Improvements in allergic rhinitis symptoms have been reported as well, likely due to reduced eosinophilic inflammation across mucosal surfaces [15].

Furthermore, beyond treating allergic inflammation, there is growing interest in dupilumab’s potential role in modulating neurodevelopmental outcomes. A recent study reported reduced rates of ADHD medication use in children treated with dupilumab, especially among the youngest patients, suggesting that immune modulation might indirectly benefit cognitive and behavioral function [16,17]. While more longitudinal studies are needed, these findings highlight dupilumab’s expanding therapeutic potential in early life.

Despite the availability of an effective and well-tolerated systemic therapy, access to dupilumab for young children is often delayed or inconsistent [18]. Pediatric dermatology services remain limited nationwide, and structural barriers disproportionately affect children with public insurance, those from non-White racial or ethnic backgrounds, and families living outside major metropolitan centers [19,20]. In clinical practice, delays frequently stem from long referral wait times, requirements for prior authorizations from insurance companies, step therapy mandates, and variability in prescriber familiarity with dupilumab use in very young children [21].

Given these challenges, important knowledge gaps remain regarding both the clinical and system-level factors that shape care for this vulnerable population. Specifically, the high rates of comorbid atopic and neurodevelopmental conditions in young children with severe AD remain under-characterized, and little is known about how access to biologic therapy varies by patient or provider factors. This study focuses on a real-world, clinically meaningful cohort, children aged 0–5 years with moderate-to-severe AD, and seeks to (1) define their neurocognitive comorbidities, along with demographic characteristics and atopic conditions, using detailed diagnostic data, and (2) assess whether time to treatment access varies by patient factors (e.g., age, insurance type) or prescriber specialty. By addressing both the clinical and health system dimensions of early AD management, this study provides novel insights into a vulnerable pediatric population and highlights opportunities for earlier, more equitable intervention.

## 2. Materials and Methods

We conducted a retrospective chart review of children who were prescribed dupilumab at or before age 5 years within the Massachusetts General Brigham (MGB) system between January 2022 and January 2024. Institutional Review Board (IRB) approval (number) was obtained prior to study initiation. Restricting the cohort to children under age five was intentional, as this developmental window represents a period of rapid neurocognitive growth when systemic inflammation may have outsized consequences. AD was the only FDA-approved condition for the use of dupilumab in children <5 years of age during the study period. Therefore, we excluded patients with primary indications listed that only included asthma or EOE from the analysis of delays in access.

The demographic variables collected included age at initiation (in months), sex, race, ethnicity, and insurance type. Clinical data included AD diagnosis, presence of atopic comorbidities (asthma, EOE, and food allergies) and neurodevelopmental diagnoses. All diagnoses were defined using ICD-10 codes. Atopic conditions included atopic dermatitis (ICD-10 L20), allergic rhinitis (ICD-10 J30.9), asthma (ICD-10 J45), eosinophilic esophagitis (ICD-10 K20.0), and food allergy (ICD-10 Z91.01). Neurodevelopmental comorbidity was defined broadly to capture the full range of relevant diagnoses and included developmental delay (ICD-10 R62.0), global developmental delay (ICD-10 Z13.42), autism spectrum disorder (ICD-10 F84), ADHD/ADD (ICD-10 F90), gross motor delay (ICD-10 F82), intellectual disabilities (ICD-10 F70, F71, F72, F73, F78, F79), speech and language disorders (ICD-10 F80), specific developmental disorders of scholastic skills (ICD-10 F81), mild cognitive disorder (ICD-10 F06.7), speech disturbances (ICD-10 R47), and dyslexia (ICD-10 R48).

Access to dupilumab was quantified by the duration in days between the dates of initial prescription and insurance approval. Prescriber specialty was categorized as dermatology or other (including allergy/immunology, gastroenterology, and pulmonology). All data were analyzed using SPSS statistics (v29). One-way ANOVAs were used to assess differences in time to treatment initiation across groups, which were defined by sex, race, ethnicity, insurance type, prescriber specialty, EOE, asthma, and food allergies. Chi-squared tests were used to compare the likelihood of having a neurodevelopmental delay across patient subgroups. Statistical significance was set at *p* < 0.05.

## 3. Results

### 3.1. Cohort Characteristics

A total of 79 children were identified aged 0–5 years at the initiation of dupilumab with an average age of 3.39 years (40.66 months). The cohort was predominantly male (62.03%), consistent with prior epidemiologic reports suggesting that AD in early childhood occurs more frequently in boys, although this sex difference often diminishes or reverses during adolescence [22]. The cohort was also racially and ethnically diverse, with 48.10% identifying as White, 25.32% as Black, 16.46% as Hispanic/Latino, and 10.13% as Asian or Other/Unknown (Table 1). Nearly half of the children (48.10%) were insured by public programs or had no insurance coverage, while the remainder had private insurance.

### 3.2. Atopic Comorbidities

The burden of atopic disease was substantial in this cohort. Food allergy was documented in 64.56%, asthma in 34.18%, and EOE in 10.13%. Almost three fourths of children (73.42%) carried two or more atopic diagnoses, and 30.38% carried two or more. Notably, 6.33% of the cohort had comorbid AD, EOE, asthma, and food allergies.

### 3.3. Neurocognitive Comorbidities

Neurodevelopmental comorbidity was also prevalent in this cohort; 43.04% of children carried at least one neurodevelopmental diagnosis, compared with an estimated national prevalence of 8–10% reported by the CDC. Across subcategories, the most frequent were speech and language disorders (25.32%), followed by developmental delay (11.39%), autism spectrum disorder (6.33%), ADHD/ADD (3.80%), and motor delay (1.27%). Notably, individuals with EOE displayed a significantly higher prevalence of neurodevelopmental diagnoses compared to those without EOE (87.50% versus 38.03%, *p* < 0.01, chi-squared; Table S1). There were no significant differences in the prevalence of developmental delay by sex, race, or insurance type.

### 3.4. Time to Treatment Approval

Of the 79 children, 56 had clearly delineated timepoints for initial insurance prior -authorization submission and approval dates with indication of or including AD; these children were included in medication access time analysis. Across prescriber specialties, dermatologists comprised 70.89% of the prescribers, with allergists, gastroenterologists, and pulmonologists constituting the remaining 29.11% (Table 1).

The overall average time to treatment approval was 20.95 weeks. Approval times differed significantly by prescriber specialty. Dermatologists achieved a mean time to approval of 10.62 ± 1.86 days compared with 51.93 ± 18.04 days for other specialists (*p* < 0.001, one-way-ANOVA; Table 2). This disparity was even more pronounced in children under the age of 3, where the mean time to approval was 10.61 ± 2.75 days for dermatologists versus 119.75 ± 38.28 days for other prescribers (*p* < 0.001; two-way-ANOVA).

Individuals with EOE experienced significantly longer times to approval compared with those without EOE (61.00 ± 32.98 days vs. 16.14 ± 4.06 days, *p* = 0.001). Sex, gender, ethnicity, and insurance type were not associated with differences in approval times. Other medical comorbidities such as asthma and food allergies did not significantly affect approval duration.

## 4. Discussion

Our study provides a detailed look at a very young pediatric population initiating dupilumab, many of whom carried a substantial comorbidity burden. Notably, 43.04% of children in our cohort had a diagnostic code for a neurodevelopmental condition prior to treatment (over four times the national average), with this prevalence rising to 87.50% in those with both EOE and AD. This observation aligns with prior literature on the intersection of atopic and neurocognitive conditions but also indicates specific prevalence rates in young children with moderate-to-severe AD, which have been infrequently reported [8,9]. This stark elevation suggests that children with early, severe AD may represent a particularly high-risk subgroup that warrants closer monitoring, raising questions about whether current screening practices adequately capture developmental vulnerabilities in this population, or whether delays remain under-recognized.

Recognizing this association is important because developmental delays in early childhood can have lasting effects on academic performance, social functioning, and quality of life [23,24]. Greater awareness in certain groups by clinicians and caregivers can prompt earlier screening and lead to timely referrals for supportive interventions [24]. Making this link more widely known could also help drive research priorities and inform coverage policies to support early biologic intervention for appropriately selected patients.

Our results build on existing evidence. A large Korean birth cohort found that children diagnosed with AD before age two were at significantly increased risk for neurodevelopmental dysfunction when assessed at age six, particularly in the gross and fine motor domains. The risk was further elevated among children who were hospitalized or treated with systemic corticosteroids, suggesting that disease severity plays a role in developmental outcomes [25]. Similarly, a U.S. population-based study using NHIS 2021 data found that children with AD were more likely to experience caregiver-reported difficulties with learning and memory, particularly in those with co-occurring ADHD or learning disabilities [17]. While these studies provide valuable insights into the long-term developmental risks associated with AD, they rely on broad pediatric age ranges and do not focus specifically on children under age five receiving systemic treatment.

Early-life Th2-driven inflammation is increasingly recognized as a key mechanistic link between AD and neurodevelopmental alterations. Excess signaling through interleukin (IL)-4 and IL-13 not only drives systemic immune dysregulation but also alters gut microbiome composition. These shifts weaken blood–brain barrier (BBB) integrity and facilitate the infiltration of cytokines into systemic circulation and, ultimately, the central nervous system (CNS). In children, the developing cerebral vasculature is particularly vulnerable to such cytokine infiltration, amplifying the potential for inflammatory injury during critical windows of brain development [8].

Within the CNS, microglia and astrocytes, the resident immune and support cells, play essential roles in shaping synaptic connectivity, supporting neurogenesis, and maintaining BBB function [8]. During early developmental periods, these cells are highly sensitive to cytokine signaling. Under physiologic conditions, microglia and astrocytes prune synapses and refine neuronal circuits to promote optimal connectivity. However, in an inflammatory environment, they shift toward pro-inflammatory phenotypes that impair white matter integrity, disrupt hippocampal development, and suppress neurogenesis. The hippocampus, a structure central to memory and learning, appears particularly vulnerable [8,26].

Animal studies have demonstrated that early-life overexposure to IL-4 reduces myelination, leading to cognitive deficits and developmental delays [26]. Parallel evidence from children with autism spectrum disorder has shown elevated levels of pro-inflammatory cytokines in both blood and brain tissue, associated with microglial and astrocytic activation, hypomyelination, and altered functional connectivity [27]. These convergent findings underscore the biologic plausibility of the elevated rates of developmental delay, ADHD, and other neurocognitive impairments observed in children with severe AD.

Another important dimension to consider is the impact of sleep disruption on neurocognitive function. A recent systematic review found that atopic dermatitis is consistently associated with impaired sleep efficiency, which in turn correlates with short-term neurocognitive deficits such as poor focus, attention, and even lower IQ scores in pediatric patients [28]. Although long-term cognitive decline remains less certain, these findings underscore that sleep quality is a potentially modifiable mediator between AD and neurodevelopmental outcomes.

Dupilumab offers a way to rapidly reduce Th2-driven inflammation by blocking the main cytokines involved in AD. A recent study highlighted the significant benefits of dupilumab treatment, showing a reduced risk of new-onset atopic and neurocognitive comorbidities, with particularly strong effects observed in younger children [29]. Notably, AD shares pathophysiological similarities with ADHD, and children with ADHD often present with atopic conditions up to two years before an ADHD diagnosis [30,31,32]. These findings raise the possibility that early initiation of target therapy during this sensitive developmental period could have implications beyond skin disease, though this theory remains to be confirmed through longitudinal research [29].

Within this context, timely medication access is especially important. In our cohort, dermatologists often secured faster access to dupilumab compared with other specialties. This is not unexpected, as AD is primarily managed by dermatologists, who are typically more familiar with dupilumab prescribing requirements and may be perceived by insurers as the most appropriate specialists to initiate therapy. For some patients, various specialists collaborated to identify which provider would have most success in prior authorization approval if more than one atopic condition was involved. Notably, individuals with EOE experienced longer approval times. Differences in insurance authorization processes and uncertainty surrounding the use of dupilumab for overlapping atopic indications in very young children may have also contributed to these delays. These findings should encourage inter-specialty collaboration to optimize access to dupilumab with the knowledge that atopic conditions tend to occur comorbidly. Positioning pediatric dermatologists within multispecialty clinics or leveraging shared electronic medical record systems for coordinated prior authorization may improve efficiency. Likewise, cross-specialty education on biologic prescribing in children under five years old could reduce variability in provider familiarity and empower more clinicians to initiate therapy when appropriate.

In addition to its efficacy, dupilumab has demonstrated a consistently favorable safety profile in pediatric populations, including infants as young as six months, with conjunctivitis as the most common side effect [33]. Despite this, real-world access to biologic therapy remains limited. In the U.S., children from Black and Hispanic backgrounds are disproportionately affected by moderate-to-severe AD, yet remain underrepresented in clinical trials and frequently experience longer delays in accessing specialty care [34]. Studying these disparities is essential, as structural barriers not only influence the timeliness of biologic initiation but may also widen developmental and health outcome gaps in vulnerable populations.

Limitations include the retrospective, non-interventional nature of this study, which introduces the possibility of incomplete or inconsistent documentation in the electronic health record, and reliance on ICD-10 codes may lead to under- or over-estimation of the true prevalence of atopic and neurodevelopmental comorbidities, as coding practices can vary between providers and over time. Because our cohort primarily included children with moderate-to-severe atopic dermatitis eligible for systemic therapy, patients with milder disease were underrepresented, and the absence of standardized severity measures such as SCORAD limited our ability to compare comorbidity burden across the full spectrum of disease severity. The relatively small cohort size and the localization to a single healthcare organization (encompassing Massachusetts General Hospital, Brigham and Women’s (BWH), BWH Faulkner, and Cooley Dickinson Hospital) limit statistical power and increase the possibility of Type II errors. Moreover, the predominance of an academic tertiary-care setting may also bias the cohort toward children with more severe disease or greater access to subspecialty care than might be observed in community settings.

Additional research is necessary to better characterize the neurodevelopmental impact of dupilumab and the relationship between atopic dermatitis severity and comorbidity burden in this young atopic population. Our findings should be viewed as hypothesis-generating, and they underscore the need for prospective, multi-center studies with larger and more socioeconomically diverse populations. Future large prospective studies incorporating standardized developmental assessments, such as academic performance and social functioning, both before and after treatment, could help inform whether early dupilumab initiation alters such long-term outcomes. Integration of caregiver-reported outcomes and health-related quality-of-life instruments would also provide a more comprehensive understanding of the real-world impact of early dupilumab treatment. Future studies should also investigate caregiver perspectives, as the decision to start biologic therapy in very young children is often influenced by parental concerns regarding safety, logistics, and cost. Qualitative research could identify the psychosocial and financial stressors that families face during the approval process, offering insights into interventions that extend beyond clinical efficacy.

In conclusion, our study highlights a high prevalence of both atopic and neurodevelopmental comorbidities among young children with moderate-to-severe atopic dermatitis. Delays in dupilumab approval were observed, particularly among those with eosinophilic esophagitis and non-dermatology providers, underscoring the importance of improved coordination across specialties. These findings highlight the need for early recognition, equitable access, and continued research to optimize outcomes for young children with severe atopic dermatitis during this critical developmental window.

## Figures and Tables

**Table 1:** Demographic characteristics of children aged 0–5 years prescribed dupilumab, *n* = 79.

Characteristics	Mean (SEM)
Age (M)	40.66 (1.75)
Characteristics	%N
Legal Sex	
Female	37.97%
Male	62.03%
Race	
White	48.10%
Black	18.99%
Asian	10.13%
Other	22.78%
Ethnicity	
Hispanic	22.78%
Non-Hispanic or unavailable	77.22%
Insurance	
Private	51.90%
Public or none	48.10%
Comorbidities	
Atopic dermatitis	98.73%
Asthma	34.18%
Eosinophilic esophagitis	10.13%
Food allergies	64.56%
Developmental or speech delay	43.04%
Provider Specialty	
Dermatology	70.89%
Other ^1^	29.11%

*SEM* = standard error of mean. ^1^ Pulmonogy, gastroenterology, allergy, and immunology.

**Table 2:** Mean time to dupilumab approval by patient characteristics and prescriber specialty.

Variable	Mean Time-Lapse (SEM) in Days	*p*-Value ^1^
Legal Sex		0.68
Female	18.27 (7.59)	
Male	22.68 (7.08)	
Race		0.46
Black	11.54 (2.83)	
White	17.04 (6.86)	
Asian	34.00 (23.38)	
Unavailable or other	31.33 (14.81)	
Ethnicity		0.24
Hispanic	9.69 (2.94)	
Non-Hispanic or unavailable	24.35 (6.64)	
Insurance		0.61
Private	23.81 (8.47)	
Public	18.47 (6.44)	
Prescriber Specialty		<0.001
Dermatology	10.62 (1.86)	
Allergy/GI/pulmonology	51.93 (18.04)	
EOE		0.01
Yes	61.00 (32.98)	
No	16.14 (4.06)	
Asthma		0.67
Yes	23.81 (10.31)	
No	19.23 (5.67)	
Food Allergies		0.10
Yes	27.51 (7.97)	
No	10.00 (2.88)	

GI = gastroenterology. ^1^ One-way ANOVAs.

## Data Availability

The raw data supporting the conclusions of this article will be made available by the authors on request.

## Supplementary Materials

The following supporting information can be downloaded at: https://www.mdpi.com/article/10.3390/children12121639/s1. Table S1: Characteristics and proportions of patients with developmental delays.

## Abbreviations

The following abbreviations are used in this manuscript:

AD	Atopic dermatitis
EOE	Eosinophilic esophagitis
ADHD	Attention-deficit/hyperactivity disorder
ADD	Attention-deficit disorder
MGB	Massachusetts General Brigham
GI	Gastroenterology
BWH	Brigham and Women’s Hospital
