# Supplementary material for: Retrospective Study of Severe Atopic Disease in Young Children (0–5 Years) Managed with Dupilumab Highlights Significant Comorbidities
Source: Children (Basel). 2025 Dec 2;12(12):1639. doi: 10.3390/children12121639 (PMC12731605; doi:10.3390/children12121639)
Supplement: Supplementary file 1 [file children-12-01639-s001.zip › children-3906999-supplementary.pdf]

Table S1. Characteristics and proportions of patients with developmental delays.

Characteristic	Proportions	P-value[1]
**Legal Sex**		0.17
Female	33.33%	
Male	48.98%	
**Race**		0.07
Black	53.33%	
White	44.74%	
Asian	0.00%	
Unavailable/other	50.00%	
**Ethnicity**		0.22
Hispanic	55.56%	
Non-Hispanic or unavailable	39.34%	
**Insurance**		0.23
Private	36.59%	
Public	50.00%	
**Comorbidities**		
Atopic dermatitis	42.31%	0.25
EOE	87.50%	**0.01**
Asthma	48.15%	0.51
Food allergies	39.22%	0.35

[1]Chi-squared test.
